# It Is about Quality, Not the Badge: A Response to Is the Ph.D. Well for Nursing Faculty Running Dry?

**DOI:** 10.3390/nursrep11040070

**Published:** 2021-09-28

**Authors:** Martin Jones, Alison Bone, Julia Heffernan

**Affiliations:** 1 Department of Rural Health, Allied Health and Human Performance, Whyalla Campus, University of South Australia, 111 Nicolson Avenue, Whyalla, SA 5608, Australia; 2 School of Nursing and Midwifery, La Trobe University, Melbourne, VIC 3086, Australia; 20199676@students.latrobe.edu.au (A.B.); Julia.Heffernan@act.gov.au (J.H.)

We read with interest the editorial by Watson et al. (2021) [1], who argue that the continued low number of nurses without a Ph.D. threatens the academic integrity of nursing programs in universities. As a consequence, they suggest that this will seriously inhibit nurses’ abilities to conduct quality research. Watson and colleagues [1] are correct: nurses do require training to conduct high quality research. However, we challenge what seems to be the central tenant of the editorial: the Ph.D. is the only training pathway that can equip nurses with the skills to conduct quality research. Furthermore, Watson outlined in 2021 outline a number of arguments to support their case [1], namely:

1. Nothing is equivalent to a Ph.D.
2. Nursing Ph.D. programs produce nurses who produce quality research.
3. Other disciplines would not support the conduct of research without a Ph.D.

In this editorial, we want to respond to Watson and colleagues [1] and present the argument that professional doctorate programs can produce nurses who are research credible, and who can produce research that is original, rigorous, and clinically meaningful. 

## 1. Nothing Is Equivalent to a Ph.D.

Professor Mark Hayter, one of Watson’s co-authors, proclaimed on *twitter*, “We say there is nothing ‘equivalent to a Ph.D.’ in our latest editorial…” [2]. Such a provocative statement does beg the question: Are all Ph.D.’s equal? Such an elitist argument leads to other equally indulgent debates: Is a Ph.D. from a Russel Group or Sandstone University equal to one from a former “Poly”? University “Top Trumps” is a silly game nurses would be well advised to avoid. Ph.D. or Professional Doctorate, Sandstone Ph.D. or Poly Ph.D.; these arguments are about labels, not substance. Our relentless concentration as a discipline should be on how we enable nurses to undertake high quality, impactful research that benefits patients. We suggest that our focus should be on the quality of the research output and not the badge of the author, and that requires that we put our heads down and get on with the work. Collectively, as professional doctorate trained researchers, we have held National Institute for Health Research (NIHR) grants in England; worked with academics in the UK, Australia, China, and the European Union; undertaken randomized controlled trials and systematic reviews; created hypothesis through interviewing patients, families, and health care workers; and generated over USD 4 million in research grant funding through conducting research training while still in practice. If the *nothing is equivalent to a Ph.D.* argument is correct, it might be helpful if they provided some evidence to substantiate such a claim; after all, the plural of anecdote is not evidence! For example, perhaps the authors could compare the h-index of researchers with a Ph.D. to those with a professional doctorate? Watson and colleagues have pedigree in this field. Reporting the h-indexes of professors of nursing in the UK in 2017, Watson and colleagues [3] observed that the median h-index score was 12. For the record, one of the author’s h-Index of this editorial was 17. 

## 2. Nursing Ph.D. Programs Produce Nurses Who Produce Quality Research

Impactful research needs to be both novel and methodologically rigorous. Watson and colleagues argued that the nurses with a Ph.D. acquire essential research skills. However, there is good evidence that this does not produce nurses who conduct methodologically rigorous research. For example, in a centimetric study of 137 qualitative studies, Walsh et al. [4] examined the quality reporting of qualitative research, a paradigm in which nurses claim to excel. The reporting of this paradigm was below the required standard. A similar picture emerges in the reporting of nursing clinical trials, the highest form of evidence that influences clinical practice. Gray and colleagues in 2017, for example, demonstrated that many trials do not meet basic reporting requirements [5]. In both of these examples, studies were conducted by nurses wearing the Ph.D. badge. 

## 3. Other Disciplines Would Not Do This

A key argument in Watson et al’s editorial [1] is that other disciplines would not employ academics to conduct research without a Ph.D., stipulating that they “would find this concept inconceivable”. In our experience, this is not correct. We have much to learn from doctors. Medics are able to retain clinical credibility and conduct research as part of their clinical role. There are numerous global examples of medical colleagues producing ground-breaking clinical research that changes clinical practice and transforms how we educate the future workforce. 

## 4. Professional Doctorates to Address Nursing Ph.D. Shortcomings

Professional doctorates provide opportunities for clinically credible nurses to be trained to conduct high quality research, and practice real world research, as part of their role. The professional doctorate exposes students to interprofessional research training, broadening their exposure to a range of study designs and methods. Typically, nurses study for their professional doctorate while retaining their clinical role and incorporate the research component of the professional doctorate into their clinical role. This provides an opportunity for nurses to extend their influence and reach in an industry focused research program. The strength of the professional doctorate is that it prepares clinicians to conduct clinically meaningful research while they are embedded in a clinical practice. Could a nursing professional doctorate program address the methodological shortcomings of the nursing research we have described? We would assert that it does; this leads us to conclude that nursing research does not require more Ph.D.s. Rather, what is needed is an agenda for more rigorous research and opportunities to conduct research. As proud professional doctorate nurses, we would argue that this research award is an important way to build research capacity within the nursing discipline. We made an informed decision to follow the professional doctorate in a nursing pathway as we worked in a clinical practice. The program allowed us to build skills and work for the benefit of our clinical areas. In many ways, we view professional doctorates as serving a similar role to the medical doctorate or psychology doctorate qualification, providing on the job research training that benefits patients. As a supervisor of nursing Ph.D. students in Australia, a nursing Ph.D. program takes the clinician from clinical practice, unlike the professional doctorate, which further embeds the clinician in a practice to conduct meaningful research. We further assert that we have seen too many nursing Ph.D. doctorate research programs where the benefit to practice is hard to discern. Realistically speaking, how much more research do we need that is far removed from the clinical setting? 

In summary, Watson and his colleagues have argued that a nursing Ph.D. program produces nurses who conduct quality research [1]. To date, there is little evidence that a nursing Ph.D. program delivers this. We would argue that a professional doctorate program embedded in a clinical practice may be the avenue to clinically engaged nursing researchers who have the ability to translate their research into clinical practice. 

## Data Availability

Not applicable.
